# Supplementary material for: Pro-SMP finder–A systematic approach for discovering small membrane proteins in prokaryotes
Source: PLoS One. 2024 Feb 29;19(2):e0299169. doi: 10.1371/journal.pone.0299169 (PMC10903887; doi:10.1371/journal.pone.0299169)
Supplement: S1 File — (DOCX) [file pone.0299169.s001.docx]

**SUPPLEMENTARY DATA**

**Supplementary Table 1**. Negative controls were taken using the EcoCyc search filters, using the following settings: sequence length (15-180 amino acids), proteins only, cell component being cytosolic, remove any that are also labeled as membrane. Positive controls were taken using the EcoCyc search filters, using the following settings: sequence length (15-180 amino acids), proteins only, protein feature being transmembrane, remove any that are not listed in the NCBI reference genome assembly. Those which were misclassified are at the end of each list in italics. For the full testing set and the best settings for options, the software has a false positive rate of 5.8% and a false negative rate of 2%. Note that the testing sets were chosen for a fairly wide range of lengths to have a larger testing set. Our testing data and scripts are contained in the Gitlab repository for the project.


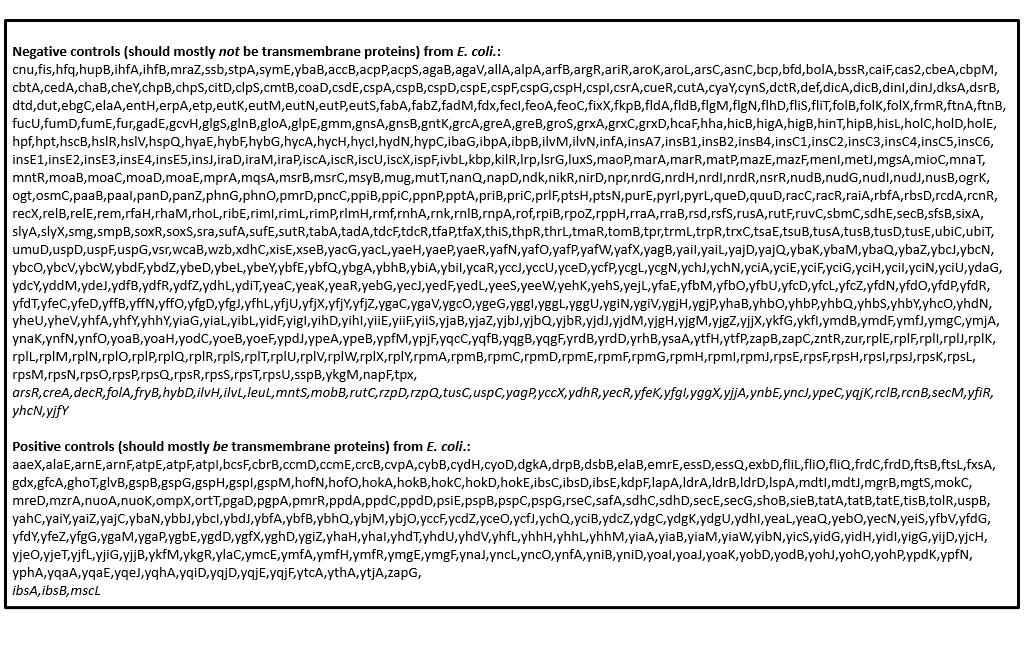


**Supplementary Table 2**. The genome information of bacteria and archaea analyzed in this study. For each microorganism, the following are given: genome assembly used, number of coding sequences (CDS) that were predicted to be SMPs (those <= 60 amino acids), and number of potential SMPs found within intergenic regions and being <= 60 amino acids.

| **Microorganism** | **Genome Assembly** | **CDS <= 60 AA** | **Inter-genic <= 60** |
| --- | --- | --- | --- |
| **Gram-positive bacteria** | | | |
| *Actinomyces* sp. oral taxon 414 | GCF_001278845.1_ASM127884v1 | 2 | 4 |
| *Aerococcus urinae* ACS-120-V-Col10a | GCF_000193205.1_ASM19320v1 | 1 | 3 |
| *Arthrobacter* sp. FB24 | GCF_000196235.1_ASM19623v1 | 7 | 8 |
| *Bacillus subtilis* | GCF_000009045.1_ASM904v1 | 26 | 19 |
| *Caldicellulosiruptor saccharolyticus* | GCF_000016545.1_ASM1654v1 | 1 | 9 |
| *Clostridioides difficile* 630 | GCF_000009205.2_ASM920v2 | 10 | 9 |
| *Clostridium acetobutylicum* ATCC 824 | GCF_000008765.1_ASM876v1 | 8 | 15 |
| *Companilactobacillus ginsenosidimutans* | GCF_001050475.1_ASM105047v1 | 2 | 8 |
| *Cutibacterium acnes* KPA171202 | GCF_000008345.1_ASM834v1 | 5 | 4 |
| *Deinococcus radiodurans* | GCF_000008565.1_ASM856v1 | 1 | 3 |
| *Desulfitobacterium hafniense* Y51 | GCF_000010045.1_ASM1004v1 | 3 | 12 |
| *Enterococcus faecalis* V583 | GCF_000007785.1_ASM778v1 | 12 | 12 |
| *Lacticaseibacillus paracasei* ATCC | GCF_000014525.1_ASM1452v1 | 16 | 7 |
| *Lactobacillus acidophilus* NCFM | GCF_000011985.1_ASM1198v1 | 5 | 10 |
| *Lactococcus lactis* subsp. lactis Il1403 | GCF_000006865.1_ASM686v1 | 5 | 1 |
| *Leuconostoc mesenteroides* subsp. mesenteroides ATCC 8293 | GCF_000014445.1_ASM1444v1 | 6 | 1 |
| *Limosilactobacillus fermentum* F-6 | GCF_000397165.1_ASM39716v1 | 6 | 24 |
| *Listeria monocytogenes* EGD-e | GCF_000196035.1_ASM19603v1 | 1 | 5 |
| *Microbacterium testaceum* | GCF_000202635.1_ASM20263v1 | 7 | 4 |
| *Nocardia farcinica* IFM 10152 | GCF_000009805.1_ASM980v1 | 11 | 11 |
| *Oenococcus oeni* | GCF_020510245.1_ASM2051024v1 | 4 | 9 |
| *Prochlorococcus marinus* subsp. marinus CCMP1375 | GCF_000007925.1_ASM792v1 | 13 | 4 |
| *Rhodococcus jostii* | GCF_000014565.1_ASM1456v1 | 12 | 9 |
| *Staphylococcus aureus* subsp. aureus NCTC 8325 | GCF_000013425.1_ASM1342v1 | 8 | 13 |
| *Streptococcus mutans* UA159 | GCF_000007465.2_ASM746v2 | 8 | 10 |
| *Streptococcus pneumoniae* TIGR4 | GCF_000006885.1_ASM688v1 | 8 | 14 |
| *Streptococcus pyogenes* | GCF_000422045.1_ASM42204v1 | 7 | 4 |
| *Streptomyces coelicolor* | GCF_000203835.1_ASM20383v1 | 16 | 43 |
| *Synechococcus elongatus* PCC6301 | GCF_000010065.1_ASM1006v1 | 28 | 2 |
| *Thermoanaerobacter* sp. X514 | GCF_000019065.1_ASM1906v1 | 6 | 11 |
| *Vagococcus teuberi* | GCF_001870205.1_ASM187020v1 | 9 | 10 |
| *Veillonella parvula* | GCF_000024945.1_ASM2494v1 | 1 | 6 |
| *Weissella koreensis* | GCF_000219805.1_ASM21980v1 | 3 | 1 |
| **Gram-negative bacteria** | | | |
| *Acetobacter pasteurianus* | GCF_000723785.2_AP1 | 5 | 9 |
| *Acinetobacter baumannii* | GCF_000786735.1_ASM78673v1 | 13 | 25 |
| *Aeromonas hydrophila* | GCF_001895965.1_ASM189596v1 | 13 | 35 |
| *Alteromonas mediterranea* | GCF_000439515.1_ASM43951v1 | 8 | 14 |
| *Bacteroides thetaiotaomicron* 7330 | GCF_001314975.1_ASM131497v1 | 10 | 29 |
| *Bacteroides thetaiotaomicron* VPI-5482 | GCF_000011065.1_ASM1106v1 | 8 | 32 |
| *Bdellovibrio* | GCF_006874645.1_ASM687464v1 | 2 | 0 |
| *Bifidobacterium longum* NCC2705 | GCF_000007525.1_ASM752v1 | 2 | 8 |
| *Bordetella pertussis* | GCF_000812165.1_ASM81216v1 | 16 | 3 |
| *Borreliella burgdorferi* B31 | GCF_000008685.2_ASM868v2 | 2 | 7 |
| *Brucella abortus* | GCF_000054005.1_ASM5400v1 | 12 | 35 |
| *Brucella melitensis* | GCF_000007125.1_ASM712v1 | 13 | 33 |
| *Buchnera aphidicola* | GCF_000007725.1_ASM772v1 | 0 | 0 |
| *Burkholderia mallei* | GCF_000755785.1_ASM75578v1 | 17 | 22 |
| *Campylobacter jejuni* | GCF_000009085.1_ASM908v1 | 5 | 1 |
| *Chlamydia trachomatis* D/UW-3/CX | GCF_000008725.1_ASM872v1 | 0 | 0 |
| *Corynebacterium glutamicum* ATCC 13032 (Kyowa Hakko) | GCF_000011325.1_ASM1132v1 | 3 | 2 |
| *Coxiella burnetii* | GCF_000007765.2_ASM776v2 | 4 | 8 |
| *Desulfovibrio vulgaris* | GCF_000015485.1_ASM1548v1 | 6 | 3 |
| *Escherichia coli* | GCF_000005845.2_ASM584v2 | 41 | 107 |
| *Francisella tularensis* | GCF_000008985.1_ASM898v1 | 4 | 2 |
| *Fusobacterium nucleatum* subsp. nucleatum ATCC 25586 | GCF_000007325.1_ASM732v1 | 3 | 19 |
| *Haemophilus influenzae* | GCF_000931625.1_ASM93162v1 | 7 | 11 |
| *Helicobacter pylori* | GCF_000600125.1_ASM60012v1 | 1 | 1 |
| *Klebsiella pneumoniae* subsp. pneumoniae HS11286 | GCF_000240185.1_ASM24018v2 | 40 | 106 |
| *Legionella pneumophila* | GCF_000347615.1_ASM34761v1 | 8 | 5 |
| *Leptospira interrogans* serovar Lai 56601 | GCF_000092565.1_ASM9256v1 | 3 | 48 |
| *Mannheimia haemolytica* | GCF_000963635.1_ASM96363v1 | 5 | 9 |
| *Moraxella catarrhalis* | GCF_000766665.1_ASM76666v1 | 4 | 7 |
| *Neisseria gonorrhoeae* | GCF_000020105.1_ASM2010v1 | 5 | 22 |
| *Neisseria meningitidis* | GCF_000626595.1_ASM62659v1 | 6 | 52 |
| *Pasteurella multocida* | GCA_029649265.1_PDT001577785.1 | 6 | 5 |
| *Pseudomonas aeruginosa* | GCF_000006765.1_ASM676v1 | 9 | 12 |
| *Rickettsia prowazekii* | GCF_000277165.1_ASM27716v1 | 2 | 1 |
| *Salmonella enterica* subsp. enterica serovar Typhi str. Ty2 | GCF_000007545.1_ASM754v1 | 30 | 92 |
| *Sinorhizobium meliloti* | GCF_000346065.1_ASM34606v1 | 26 | 49 |
| *Treponema denticola* | GCF_000008185.1_ASM818v1 | 3 | 2 |
| *Vibrio cholerae* | GCF_000963555.1_ASM96355v1 | 27 | 80 |
| *Xanthomonas campestris* | GCF_000221965.1_ASM22196v1 | 7 | 22 |
| *Yersinia pestis* A1122 | GCF_000222975.1_ASM22297v1 | 8 | 111 |
| *Yersinia pseudotuberculosis* | GCF_000834295.1_ASM83429v1 | 10 | 109 |
| *Zymomonas mobilis* | GCF_000007105.1_ASM710v1 | 3 | 6 |
| **Other bacteria** | | | |
| *Mycobacterium tuberculosis* H37Rv | GCF_000277735.2_ASM27773v2 | 9 | 14 |
| *Mycolicibacterium smegmatis* MC2 155 | GCF_000767605.1_ASM76760v1 | 12 | 11 |
| *Mycoplasma genitalium* G37 | GCF_000027325.1_ASM2732v1 | 0 | 0 |
| **Archaea** | | | |
| *Desulfurococcus amylolyticus* 1221n | GCF_000020905.1_ASM2090v1 | 4 | 0 |
| *Haloarcula marismortui* | GCF_000011085.1_ASM1108v1 | 12 | 10 |
| *Methanobacterium lacus* | GCF_000191585.1_ASM19158v1 | 3 | 1 |
| *Methanococcus maripaludis* S2 | GCF_000011585.1_ASM1158v1 | 3 | 0 |
| *Methanosarcina barkeri* Fusaro | GCF_000195895.1_ASM19589v1 | 12 | 19 |
| *Nitrosopumilus maritimus* | GCF_000018465.1_ASM1846v1 | 2 | 1 |
| *Pyrobaculum aerophilum* | GCF_000007225.1_ASM722v1 | 0 | 2 |
| *Pyrococcus furiosus* DSM 3638 | GCF_000007305.1_ASM730v1 | 3 | 3 |
| *Saccharolobus solfataricus* P2 | GCF_000007005.1_ASM700v1 | 1 | 0 |
| *Sulfolobus acidocaldarius* DSM 639 | GCF_000012285.1_ASM1228v1 | 1 | 1 |
| *Thermococcus kodakarensis* | GCF_000009965.1_ASM996v1 | 4 | 4 |

**Supplementary Table 3**. Putative SMPs identified in this study.

| **Microorganism** | **Putative SMPs Identified from designated ORFs** |
| --- | --- |
| **Gram-positive bacteria** |  |
|  |  |
| *Actinomyces* sp. oral taxon 414 | Hypothetical protein  Methionine/alanine import family NSS transporter small subunit |
| *Aerococcus urinae* ACS-120-V-Col10a | Preprotein translocase subunit SecE |
| *Arthrobacter* sp. FB24 | 5 hypothetical proteins  DLW-39 family protein |
| *Bacillus subtilis* | 12 hypothetical proteins  Preprotein translocase subunit  Component of the twin-arginine pre-protein translocation pathway  Putative type I toxin; phage SPbeta  Factor required extrachromosomal elements maintenance  6 putative type I toxins  Putative spore and germination protein  Spore assembly and germination protein  Spore and germination protein  Regulator of iron homeostasis |
| *Caldicellulosiruptor saccharolyticus* | YvrJ family protein |
| *Clostridioides difficile 630* | 5 hypothetical proteins  YvrJ family protein  DUF3789 domain-containing protein  FeoB-associated Cys-rich membrane protein  Cyclic lactone autoinducer peptide |
| *Clostridium acetobutylicum* ATCC 824 | YvrJ family protein  2 putative holin-like toxins  YtxH domain-containing protein  FeoB associated Cys-rich membrane protein  2 hypothetical proteins  DUF1304 domain-containing protein |
| *Companilactobacillus ginsenosidimutans* | DUF4044 domain-containing protein  Teichoic acid D-Ala incorporation-associated protein DltX |
| [*Cutibacterium acnes* KPA171202](https://www.ncbi.nlm.nih.gov/Taxonomy/Browser/wwwtax.cgi?mode=Info&id=267747&lvl=3&lin=f&keep=1&srchmode=1&unlock) | 3 hypothetical proteins  Potassium-transporting ATPase subunit F  Methionine/alanine import family NSS transporter small subunit |
| *Deinococcus radiodurans* | Hypothetical protein |
| *Desulfitobacterium hafniense* Y51 | Hypothetical protein  FeoB-associated Cys-rich membrane protein  Cyclic lactone autoinducer peptide |
| *Enterococcus faecalis* V583 | Teichoic acid D-ala incorporation-associated protein DltX  Preprotein translocase subunit SecE  DNA-directed RNA polymerase subunit beta  DUF4044 domain-containing protein  Putative holin-like toxin  rRNA adenine methyltransferase  Type I toxin-antitoxin system Fst family toxin  Hypothetical protein  Membrane protein  50S ribosomal protein L33  DUF3042 family protein |
| *Lacticaseibacillus paracasei* ATCC | Teichoic acid D-Ala incorporation-associated protein DltX  DNA-directed RNA polymerase subunit beta  DUF4044 domain-containing protein  OadG family protein  FeoB-associated Cys-rich membrane protein  Preprotein translocase subunit SecE  Type I toxin-antitoxin system Fst family toxin  YvrJ family protein  6 putative holin-like toxin  DUF3042 family protein  Hypothetical protein |
| *Lactobacillus acidophilus* NCFM | DUF4044 domain-containing protein  YvrJ family protein  Preprotein translocase subunit SecE  Teichoic acid D-Ala incorporation-associated protein  50S ribosomal protein L33 |
| *Lactococcus lactis* subsp. lactis Il1403 | Teichoic acid D-Ala incorporation-associated protein  DUF4044 domain-containing protein  2 hypothetical proteins  DUF3042 family protein |
| *Leuconostoc mesenteroides* subsp. mesenteroides ATCC 8293 | Teichoic acid D-Ala incorporation-associated protein DltX  Preprotein translocase subunit SecE  3 putative holin-like toxins |
| *Limosilactobacillus fermentum* F-6 | FeoB-associated Cys-rich membrane protein  Preprotein translocase subunit SecE  Teichoic acid D-Ala incorporation-associated protein DltX  DUF4044 domain-containing protein  Putative holin-like toxin  DUF3042 family protein  Hypothetical protein |
| *Listeria monocytogenes* EGD-e | Preprotein translocase subunit SecE |
| *Microbacterium testaceum* | 4 hypothetical proteins  K(+)-transporting ATPase subunit F |
| *Nocardia farcinica* IFM 10152 | DUF2613 domain-containing protein  K(+)-transporting ATPase subunit F  DUF4244 domain-containing protein  4 Hypothetical proteins  DUF3099 domain-containing protein  DLW-39 family protein  Helix-turn-helix domain-containing protein |
| *Oenococcus oeni* | Preprotein translocase subunit SecE  DUF1146 family protein  LysR family transcriptional regulator  Teichoic acid D-Ala incorporation-associated protein |
| *Prochlorococcus marinus* subsp. Marinus CCMP1375 | Photosystem II reaction center protein PsbN  Photosystem II reaction center protein I  Photosystem II reaction center protein K  Cytochrome b559 subunit beta  Photosystem II reaction center protein L  Photosystem II reaction center protein T  Photosystem II reaction center protein PsbM  Photosystem I reaction center subunit IX  Cytochrome b6-f complex subunit PetG  High ligh inducible protein  Photosystem I reaction center subunit VIII  DNA-binding protein  Cytochrome b6-f complex subunit PetN |
| *Rhodococcus jostii* | DUF6131 family protein  5 hypothetical proteins  Sec-independent protein translocase subunit TatA  Potassium-transporting ATPase subunit F  DUF2613 domain-containing protein |
| *Staphylococcus aureus* subsp. aureus NCTC 8325 | 8 hypothetical proteins |
| *Streptococcus mutans* UA159 | DUF4044 domain-containing protein  Preprotein translocase subunit SecE  Teichoic acid D-Ala incorporation-associated protein DltX  Hypothetical protein  Type I toxin-antitoxin system Fst family toxin  FeoB-associated Cys-rich membrane protein  DUF3042 family protein |
| *Streptococcus pneumoniae* TIGR4 | Hypothetical protein  Teichoic acid D-Ala incorporation-associated protein DltX  DUF 4044 domain-containing protein  Type I toxin-antitoxin system Fst family toxin  Preprotein translocase subunit SecE  PspC domain-containing protein  DUF3042 family protein  50S ribosomal protein L33 |
| *Streptococcus pyogenes* | Preprotein translocase subunit SecE  Teichoic acid D-Ala incorporation-associated protein DltX  DUF4044 domain-containing protein  2 type I toxin-antitoxin system Fst family toxins  DUF3042 family protein  OadG-related small transporter subunit |
| *Streptomyces coelicolor* | 9 hypothetical proteins  SpdC protein  5 small membrane proteins  Small hydrophobic hypothetical protein |
| *Synechococcus elongatus* PCC6301 | 2 Chlorophyll a/b-binding proteins  Photosystem I reaction center subunit IX  Cytochrome b559 subunit beta  Photosystem II reaction center protein J  Photosystem II reaction center protein M  Photosystem II reaction center protein T  Ssl1498 family light-harvesting-like protein  DUF3285 domain-containing protein  Photosystem II reaction center protein PsbN  7 hypothetical proteins  Cytochrome b6-f complex subunit PetM  Photosystem I reaction center subunit VIII  Photosystem II reaction center X protein  Photosystem II reaction center protein I  High light inducible protein  Photosystem II reaction center protein Ycf12  Cytochrome b6-f complex subunit PetN  Photosystem II protein Y  Photosystem I reaction center subunit XII |
| *Thermoanaerobacter* sp. X514 | 4 hypothetical proteins  ISNCY family transposase  Preprotein translocase subunit SecE |
| *Vagococcus teuberi* | DUF4044 domain-containing protein  DNA-directed RNA polymerase subunit beta  Preprotein translocase subunit SecE  LPXTG cell wall anchor domain-containing protein  Teichoic acid D-Ala incorporation-associated protein DltX  Type I toxin-antitoxin system Fst family toxin  VanZ family protein  DUF3042 family protein  50S ribosomal protein L33 |
| *Veillonella parvula* | Type I toxin-antitoxin system Fst family toxin |
| *Weissella koreensis* | Putative holin-like toxin  Preprotein translocase subunit SecE  DUF3042 family protein |
| **Gram-negative bacteria** |  |
| *Acetobacter pasteurianus* | 4 hypothetical proteins  DUF2474 family protein |
| *Acinetobacter baumannii* | 10 hypothetical proteins  Methionine/alanine import family NSS transporter small subunit  Entericidin A/B family lipoprotein  Cytochrome bd-I oxidase subunit CydX |
| *Aeromonas hydrophila* | 5 hypothetical proteins  CcoQ/FixQ family Cbb3-type cytochrome c oxidase assembly chaperone  TIGR02808 family protein  Cytochrome bd-I oxidase subunit CydX  DUF3149 domain-containing protein  Twin-arginine translocase TatA/TatE family subunit  Lipoprotein |
| *Alteromonas mediterranea* | Efflux RND transporter permease subunit  Cbb3-type cytochrome c oxidase subunit 3  3 hypothetical proteins  Entericidin A/B family lipoprotein  Cytochrome bd-I oxidase subunit CydX |
| *Bacteroides thetaiotaomicron* 7330 | 8 smalltalk proteins  2 Hypothetical proteins |
| *Bacteroides thetaiotaomicron* VPI-5482 | 8 small talk proteins |
| *Bdellovibrio* | AAA family ATPase  Cbb3-type cytochrome oxidase assembly protein CcoS |
| *Bifidobacterium longum* NCC2705 |  |
| *Bordetella pertussis* | Putative transporter small subunit  Cytochrome oxidase small assembly protein  Cytochrome bd-I oxidase subunit CydX  Cytochrome oxidase  Flp family type IVb pilin  4 hypothetical proteins  K(+)-transporting ATPase subunit F  3 Entericidin A/B family lipoproteins  DUF4148 domain-containing protein |
| *Borreliella burgdorferi* B31 | Preprotein translocase subunit SecE  Hypothetical protein |
| *Brucella abortus* | CcoQ/FixQ family Cbb3-type cytochrome c oxidase assembly chaperone  Heme exporter protein CcmD  8 hypothetical proteins  Cytochrome bd-I oxidase subunit CydX  Lipoprotein |
| *Brucella melitensis* | CcoQ/FixQ family Cbb3-type cytochrome c oxidase assembly chaperone  Heme exporter protein CcmD  9 hypothetical proteins  Cytochrome bd-I oxidase subunit CydX  Lipoprotein |
| *Buchnera aphidicola* | 2-C-methyl-D-erythritol 2,4-cyclodiphosphate synthase |
| *Burkholderia mallei* | 11 hypothetical proteins  Flp family type IVb pilin  Cytochrome bd-I oxidase subunit CydX  Entericidin A/B family lipoprotein  Lipoprotein  K(+)-transporting ATPase subunit F |
| *Campylobacter jejuni* | Protein translocase subunit SecE  Hypothetical protein  Periplasmic protein  Lipoprotein  Membrane protein |
| *Chlamydia trachomatis* D/UW-3/CX |  |
| *Corynebacterium glutamicum* ATCC 13032 (Kyowa Hakko) | 2 hypothetical proteins  Methyltransferase |
| *Coxiella burnetii* | Protein YbgT  2 hypothetical proteins  Entericidin A |
| *Desulfovibrio vulgaris* | 2 hypothetical proteins  CcmD family protein  2 Flp family type IVb pilins  Potassium-transporting ATPase subunit F |
| *Escherichia coli* | Multidrug efflux pump accessory protein AcrZ  DUF2770 domain-containing protein YceO  Protein Yoal  Pmp3 family protein YqaE  IlvXGMEDA operon leader peptide  Uncharacterized protein YncL  Small protein MgtS  Uncharacterized protein YdgU  Cytochrome bd-I accessory subunit CydH  Membrane-depolarizing toxin TisB  Putative membrane protein Yoak  Uncharacterized protein YoaJ  Putative membrane protein YohP  Putative membrane protein YpdK  Protein YmiC  Protein YecU  Small toxic polypeptide LdrA  Small toxic polypeptide LdrB  Small toxic polypeptide LdrC  Toxin HokB  Beta-lactam resistance protein  Small toxic polypeptide LdrD  Small toxic polypeptide  Uncharacterized protein YmiA  UPF0387 family protein YohO  Protein HokC  Leu operon leader peptide  K(+) transporting P-type ATPase subunit KdpF  Cytochrome bd-I ubiquinol oxidase accessory subunit CydX  Small protein Mnts  Small protein AppX  Protein YdcA  Qin prophage; toxic protein HokD  Uncharacterized protein YniD  PhoQ kinase inhibitor  Protein YqiD  DUF2556 domain-containing protein YhdU  DUF4223 domain-containing lipoprotein YhfL  Putative bitopic inner membrane protein  Entericidin A lipoprotein, antidote to entericidin B  Bacteriolytic entericidin B lipoprotein |
| *Francisella tularensis* | 3 hypothetical proteins |
| *Fusobacterium nucleatum* subsp. nucleatum ATCC 25586 | Protein translocase subunit SecE  Na(+)-linked D-alanine glycine permease  50S ribosomal protein L32 |
| *Haemophilus influenzae* | 5 hypothetical proteins  Methionine/alanine import family NSS transporter small subunit  Lipoprotein |
| *Helicobacter pylori* | Preprotein translocase subunit SecE |
| *Klebsiella pneumoniae* subsp. pneumoniae HS11286 | 20 Hypothetical Proteins  L-asparagine permease  entericidin A  entericidin B membrane lipoprotein  riboflavin synthase subunit beta  multidrug efflux pump-associated protein, AcrZ family  DUF2770 family protein  YoaK family small membrane protein  YmiA family putative membrane protein  YqaE family transport protein  transcriptional regulator  putative dimethyl sulfoxide reductase, anchor subunit  MFS transporter  DUF2474 domain-containing protein  Ecr family regulatory small membrane protein  putative outer membrane protein N precursor  small membrane protein  protein YohO  putative inner membrane protein  small toxic polypeptide  type I toxin-antitoxin system toxin TisB |
| *Legionella pneumophila* | MAPEG family protein  Heme exporter protein CcmD  3 hypothetical proteins  Cytochrome bd-I oxidase subunit CydX  Alkaline phosphatase  Lipoprotein |
| *Leptospira interrogans* serovar Lai 56601 | 2 hypothetical proteins |
| *Mannheimia haemolytica* | Cytochrome bd oxidase small subunit, CydX/CbdX family  Lipoprotein  Entericidin A/B family lipoprotein  Hypothetical protein |
| *Moraxella catarrhalis* | Methionine/alanine import family NSS transporter small subunit  Cytochrome bd-I oxidase subunit CydX  Entericidin A/B family lipoprotein  Lipoprotein |
| *Neisseria gonorrhoeae* | CcoQ/FixQ family Cbb3-type cytochrome coxidase assembly chaperone  Methionine/alanine import family NSS transporter small subunit  2 hypothetical proteins  Lipoprotein |
| *Neisseria meningitidis* | CcoQ/FixQ family Cbb3-type cytochrome c oxidase assembly chaperone  3 hypothetical proteins  Methionine/alanine import family NSS transporter small subunit  Lipoprotein |
| *Pasteurella multocida* | Entericidin A/B family lipoprotein  Cytochrome bd oxidase small subunit, CydX/CbdX family  Methionine/alanine import family NSS transporter small subunit  Nitrate/trimethylamine N-oxide reductase NapE/TorE  2 hypothetical proteins |
| *Pseudomonas aeruginosa* | Nitrate reductase protein NapE  Heme exporter protein CcmD  6 hypothetical proteins  Lipopeptide LppL |
| *Rickettsia prowazekii* | DUF2706 domain-containing protein  Sec-independent protein translocase subunit TatA |
| *Salmonella enterica* subsp. enterica serovar Typhi str. Ty2 | YnhF family membrane protein  Type I toxin-antitoxin system toxin TisB  Lipoprotein toxin entericidin B  Cytochrome bd-I oxidase subunit CydX  Cytochrome bd-II oxidase subunit CbdX  YhfL family protein  YqaE/Pmp3 family membrane protein  DUF2556 family protein  PhoP/PhoQ regulator MgrB  Acid stress response protein YqgB  LpxT activity modulator PmrR  AcrZ family multidrug efflux pump-associated protein  Protein MgtS  Entericidin A/B family lipoprotein  Stress response membrane protein YncL  DUF2633 family protein  YceO family protein  Protein YohO  Ilv operon leader peptide  Type I toxin-antitoxin system toxin Ldr family protein  K(+)-transporting ATPase subunit F  Leu operon leader peptide  Membrane protein YpdK  YoaK family small membrane protein  Division septum protein Blr  YmiA family putative membrane protein  3 hypothetical Proteins |
| *Sinorhizobium meliloti* | 2 Flp family type ivb pilins  3 ccoq/fixq family Cbb3-type cytochrome coxidase assembly chaperones  Cbb3-type cytochrome oxidase assembly protein ccos  14 hypothetical proteins  Heme exporter protein ccmd  DUF5989 family protein  Entericidin  Entericidin A/B family lipoprotein |
| *Treponema denticola* | Preprotein translocase subunit SecE  2 Hypothetical proteins |
| *Vibrio cholerae* | TIGR02808 family protein  2 Hypothetical proteins  13 DUF3265 domain-containing proteins  2 Cytochrome bd-I oxidase subunit CydX  Tfp pilus assembly protein  MetS family NSS transporter small subunit  DUF3149 domain-containing protein  CcoQ/FixQ family Cbb3-type cytochrome c oxidase assembly chaperone  YnhF family membrane protein  Cbb3-type cytochrome oxidase assembly protein CcoS  50S ribosomal protein L32 |
| *Xanthomonas campestris* | Heme exporter protein CcmD  2 hypothetical proteins  2 Entericidin A/B family lipoproteins  Potassium-transporting ATPase subunit F  Cytochrome bd-I oxidase subunit CydX |
| *Yersinia pestis* A1122 | Cytochrome bd-I oxidase subunit cydx  K(+)-transporting atpase subunit F  Ynhf family membrane protein  Acrz family multidrug efflux pump-associated protein  Ilv operon leader peptide  DUF2770 family protein  Small membrane protein ynid  Hypothetical protein |
| *Yersinia pseudotuberculosis* | 6 Hypothetical proteins  Ynhf family membrane protein acrz family multidrug efflux pump-associated protein  Ilv operon leader peptide  Acrz family multidrug efflux pump-associated protein  Cytochrome bd-I oxidase subunit cydx  K(+)-transporting atpase subunit F |
| *Zymomonas mobilis* | Hypothetical protein  Entericidin A/B family lipoprotein  DUF2474 domain-containing protein |
| **Other bacteria** |  |
| *Mycobacterium tuberculosis* H37Rv | DUF2613 domain-containing protein  DUF2752 domain-containing protein  K(+)-transporting ATPase subunit F  5 Hypothetical proteins |
| *Mycolicibacterium smegmatis* MC2 155 | 9 Hypothetical proteins  DUF6131 family protein  DUF2613 domain-containing protein |
| *Mycoplasma genitalium* G37 |  |
| **Archaea** |  |
| *Desulfurococcus amylolyticus* 1221n | Protein translocase SEC61 complex subunit gamma  Preprotein translocase subunit Sec61 beta  Hypothetical protein |
| *Haloarcula marismortui* | Preprotein translocase subunit Sec61 beta  Protein translocase SEC61 complex subunit gamma  6 Hypothetical proteins |
| *Methanobacterium lacus* | 2 class III signal peptide-containing proteins  Preprotein translocase subunit Sec61 beta  Hypothetical protein |
| *Methanococcus maripaludis* S2 | Preprotein translocase subunit Sec61 beta  Mets family NSS transporter small subunit |
| *Methanosarcina barkeri* Fusaro | Preprotein translocase subunit Sec61 beta  2 hypothetical proteins  DUF4277 domain-containing protein |
| *Nitrosopumilus maritimus* | Preprotein translocase subunit Sec61 beta  Protein translocase SEC61 complex subunit gamma |
| *Pyrobaculum aerophilum* |  |
| *Pyrococcus furiosus* DSM 3638 | 2 class III signal peptide-containing proteins  Preprotein translocase subunit Sec61 beta |
| *Saccharolobus solfataricus* P2 | Preprotein translocase subunit Sec61 beta |
| *Sulfolobus acidocaldarius* DSM 639 | Preprotein translocase subunit Sec61 beta |
| *Thermococcus kodakarensis* | Preprotein translocase subunit Sec61 beta  2 class III signal peptide-containing proteins  Hypothetical protein |
