# Supplementary material for: Pro-SMP finder–A systematic approach for discovering small membrane proteins in prokaryotes
Source: PLoS One. 2024 Feb 29;19(2):e0299169. doi: 10.1371/journal.pone.0299169 (PMC10903887; doi:10.1371/journal.pone.0299169)
Supplement: Attachment — Submitted filename: Response to reviewers PLOS ONE.pdf [file pone.0299169.s002.pdf]

Response to the editorial and reviewer's comments.

1. Please state what role the funders took in the study. If the funders had no role, please state: "The funders had no role in study design, data collection and analysis, decision to publish, or preparation of the manuscript."

- Done.

2. We notice that your supplementary tables are included in the manuscript file. Please remove them and upload them with the file type 'Supporting Information'. Please ensure that each Supporting Information file has a legend listed in the manuscript after the references list.

- Done.

3. The authors should improve the pipeline and online interface of the Pro-SMP Finder software to enhance its usability and broaden its applications.

- The items below are improved to enhance the usability and broaden its application.
    - The web interface to the pipeline has been streamlined and improved in the following ways.
    - Results can now be viewed in the web browser after the pipeline completes. Results will remain on the server for at least 2 weeks, and a download link is provided to download the results locally.
    - Results now include links to gene pages at NCBI and UniProt to facilitate further exploration.
    - Results now include an improved summary of the running of the pipeline and results from each intermediate step of the pipeline (how many genes passed each stage, how to determine where a gene was filtered out).
    - Results now include a genome browser view so that each candidate gene or intergenic region can be quickly seen in the context of other genes.
    - The listing of full results has been updated to be easier to understand.
    - The interface for the web submission form has been improved to be more intuitive and easy to use.
